# Rapid detection of feline parvovirus using RAA-CRISPR/Cas12a-based lateral flow strip and fluorescence

**DOI:** 10.3389/fmicb.2025.1501635

**Published:** 2025-03-11

**Authors:** Han Chen, Hailing Zhang, Jie Guo, Xiangshu Meng, Mengfan Yao, Longbin He, Xiaoxuan Nie, Han Xu, Chao Liu, Jian Sun, Fei Wang, Yuelong Sun, Zhong Jiang, Yanliang He, Jianlou Zhang, Jianke Wang

**Affiliations:** ^1^Hebei Veterinary Biotechnology Innovation Center, College of Veterinary Medicine, Hebei Agricultural University, Baoding, China; ^2^Key Laboratory of Special Animal Epidemic Disease, Ministry of Agriculture, Institute of Special Animal and Plant Sciences, Chinese Academy of Agricultural Sciences, Changchun, China; ^3^Weihai Ocean Vocational College, Rongcheng, China; ^4^Chongqing Three Gorges Vocational College, Chongqing, China; ^5^Agriculture Bureau of Zhuozhou City, Zhuozhou, China; ^6^Sinovet (Jiangsu) Biopharmaceuticals Co., Ltd., Taizhou, China

**Keywords:** CRISPR/Cas12a, detection, feline, parvovirus, RAA, lateral flow strip

## Abstract

Feline parvovirus (FPV) causes severe gastroenteritis and leukopenia in cats, with high morbidity and mortality, necessitating a rapid and effective antigen diagnostic test with high sensitivity and specificity. In this study, a diagnostic platform based on a combination of Recombinase-Aided Amplification (RAA) and CRISPR/Cas12a was established for detecting FPV. Cas12a recombinant protein was purified using Nickel-Nitriloacetic Acid resin after heterologous expression in *Escherichia coli*. The results of RAA-CRISPR/Cas12a can be detected with a fluorescence reader or lateral flow strips (LFS) for on-site detection. The RAA-CRISPR/Cas12a-LFS had a detection limit of 2.1 × 10^0^ copies of recombinant plasmids per reaction, compared with 2.1 × 10^3^ copies for conventional PCR analysis. Furthermore, no cross-reactivity was observed for the RAA-CRISPR/Cas12a assay with feline coronavirus, feline herpesvirus, and feline calicivirus, demonstrating reasonable specificity. Additionally, 43 cat fecal samples with suspected clinical signs were assayed with RAA-CRISPR/Cas12a-LFS and conventional PCR in parallel. The RAA-CRISPR/Cas12a-LFS showed a 100% coincident rate with PCR. In summary, a novel, visual, sensitive, and specific detection assay based on RAA and CRISPR/Cas12a was developed for FPV.

## Introduction

Feline parvovirus (FPV), also known as feline panleukopenia virus, belongs to the species *Carnivore protoparvovirus 1* of the genus *Protoparvovirus* within the family *Parvoviridae* and causes the feline panleukopenia which is a severe disease of cats, with high morbidity and mortality (Cotmore et al., 2019; Tucciarone et al., 2021). Besides domestic cats, FPV can also infect raccoons, mink, foxes, giant pandas, and monkeys. It mainly infects lymphoid tissues and intestinal epithelia in young and adult animals, leading to leukopenia or lymphopenia and severe diarrhea, vomiting, dehydration, fever, and sudden death (Yang et al., 2010; Jiao et al., 2020; Yi et al., 2021; Cao et al., 2023). FPV is a small, non-enveloped virus with a linear, single-stranded DNA genome of approximately 5.1 kb flanked by terminal palindromes (Cotmore et al., 2019). It is one of the smallest animal viruses, barely 18 to 20 nm in diameter. The genome contains two open reading frames that encode for two non-structural proteins, NS1 and NS2, and two capsid proteins, VP1 and VP2, respectively, by alternative splicing (Lin et al., 2019; Li et al., 2024). Vaccination is an essential means to control the disease. Still, vaccine failure often occurs due to stress and maternal antibodies, vaccine factors (vaccination regimens or administration), and viral variants with higher virulence (Soma et al., 2019), and the rapid and accurate detection of the virus is critical for preventing the spread of feline panleukopenia.

The World Organization for Animal Health (WOAH) has recommended several diagnostic methods for FPV, including indirect immunofluorescence assay (IFA), polymerase chain reaction (PCR), and viral isolation for identification of the agent, and antigen-capture enzyme immunoassay (EIA), antigen-capture ELISA, and haemagglutination and haemagglutination inhibition assay (HA-HI). Although those approaches have been adopted for FPV detection, they have disadvantages (Sun et al., 2019; Wang et al., 2019; Sun et al., 2021). HA-HI has been the gold standard for carnivore protoparvovirus detection. Nonetheless, HA-HI requires a continuous supply of fresh erythrocytes and is unsuitable for detecting non-haemagglutinating isolates (Wang et al., 2015; Jenkins et al., 2020). Diagnostic tools dependent on nucleic acid, including PCR, quantitative polymerase chain reaction (qPCR), and high-resolution melting (HRM) assay, have the advantage of high sensitivity for FPV detection. However, those methods are expensive, labor-intensive, time-consuming, and limited use for rapid on-site diagnosis as they require skilled technicians and well-equipped laboratories (Schunck et al., 1995; Sun et al., 2019; Wang et al., 2021).

Recombinase-aided amplification (RAA) is the most recent isothermal nucleic acid amplification technology with the recombinase, single-strand DNA binding (SSB) protein, and DNA polymerase, and it can be a point-of-care testing (POCT) due to its low resource requirements. Clustered regularly interspaced short palindromic repeats (CRISPR) and its associated proteins (Cas) exhibit an adaptive immune system guided by the RNA used by bacteria and archaea to defend against viral infections. Besides, the CRISPR/Cas system was widely used in nucleic acid detections for viruses and bacteria in clinical and animal diseases. RAA preamplification combined with the CRISPR/Cas12a detection system has been used for many viruses detection, for example, SARS-Cov-2 (Xiong et al., 2020), African swine fever (Bai et al., 2019), Monkeypox virus (Zhao et al., 2023), Maize chlorotic mottle virus (Lei et al., 2023), Severe fever with thrombocytopenia syndrome virus (Zhu et al., 2022), Human norovirus (Qian W. et al., 2022), Porcine epidemic diarrhea virus (Qian B. et al., 2022), Lumpy skin disease virus (Liao et al., 2022) and it also used for bacteria and parasites detection in some previously studies (Ma et al., 2021; Lin et al., 2022; Wang et al., 2022; Xiao et al., 2023).

**Figure 1:**
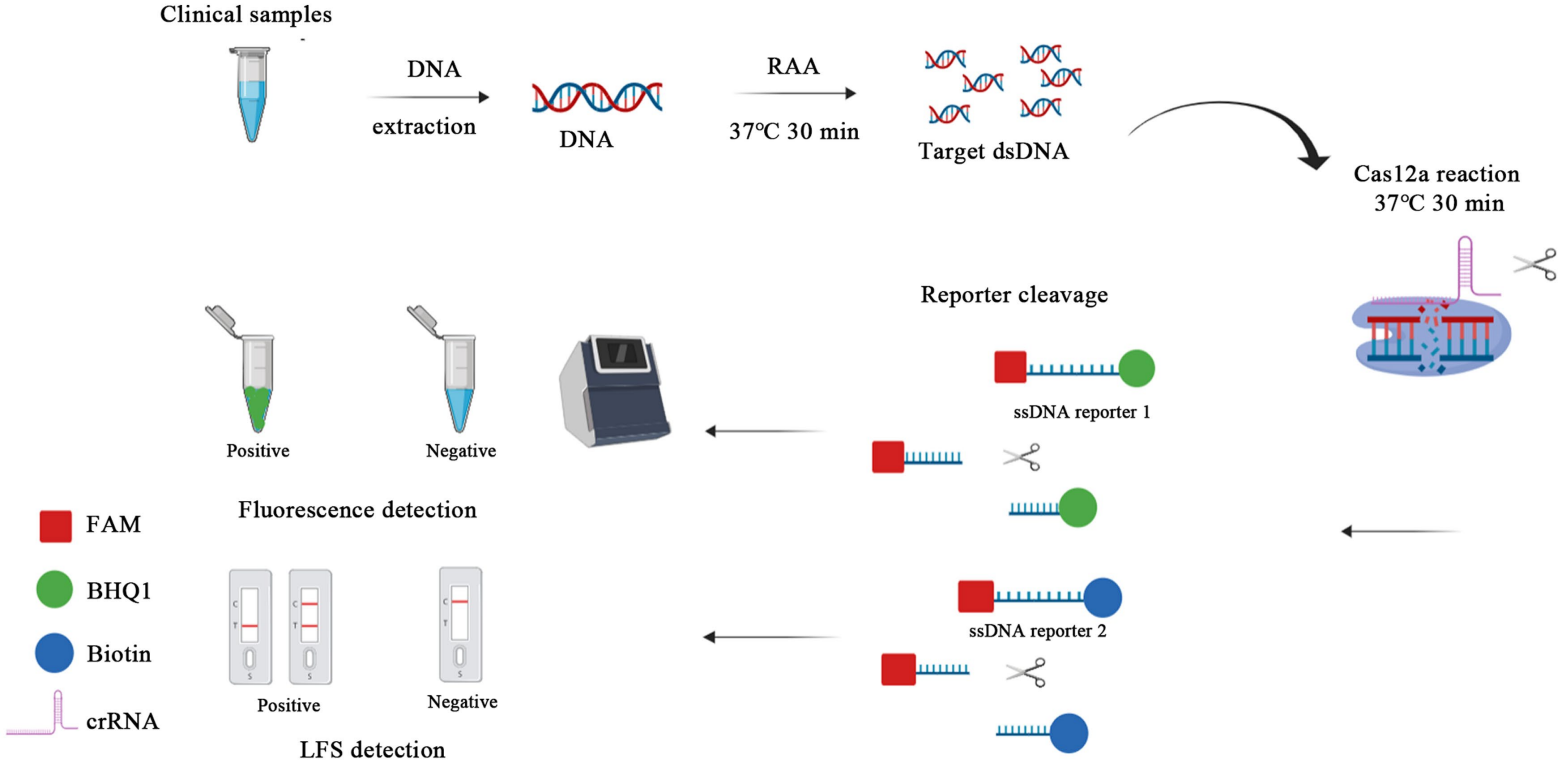
Schematic of RAA-CRISPR/Cas12a-LFS (FLUOR) diagnostic platform. Viral DNA was extracted from clinical samples and the target gene was swiftly amplified by RAA, performed at a rapid 37°C water bath for 30 min. Then the RAA products were detected by CRISPR/Cas12a-lateral flow strip (LFS) with the ssDNA reporter 2 FAM-TTTTTTTATTTTTTT-Biotin, whereas the ssDNA reporter 1 FAM-TTTATTT-BHQ1 was used for blue light, UV light, and fluorescence detection in CRISPR/Cas12a-fluorescence (FLUOR) assay.

This study aimed to develop an early rapid feline parvovirus diagnostic tool using RAA and CRISPR/Cas12a targeting the VP2 gene, a highly conserved gene among feline parvovirus. In this study, the partial VP2 gene of feline parvovirus was amplified by RAA in a dry bath at an isothermal temperature of 37°C. Then, the double-strand DNA products were cleaved by Cas12a with the help of crRNA targeting the VP2 gene site; Cas12a further cut the ssDNA probes labeled with biotin or fluorescent (Figure 1).

## Materials and methods

### Plasmids, viruses, and clinical samples

The Cas12a expression plasmid 6 × His-MBP-TEV-huLbCpf1 was a gift from Dr. Wei Ouyang in Institute of Veterinary Medicine, Jiangsu Academy of Agricultural Sciences. The DF2 strain (ATCC VR-2004) of feline coronavirus (FCoV) was purchased from ATCC and cultured in CRFK cell. The FHV BJ22-5 strain of feline herpesvirus (FHV) and the FCV BJ22-11 strain of feline calicivirus (FCV) were isolated from clinical samples in our lab. FCoV, FHV, and FCV were used to determine the specificity of the RAA-CRISPR/Cas12a/FLUOR and RAA-CRISPR/Cas12a/LFS assays. Notably, 43 fecal samples were meticulously collected from young cats suspected to be infected with feline parvovirus from animal hospitals in Qingdao, China. The sample collection procedures were approved by the Laboratory Animal Welfare and Animal Experimental Ethical Committee of Hebei Agricultural University, Baoding, China, and the animal owner consented to all the samples collected. The viral DNA/RNA were extracted from cell culture and clinical samples using a DNA/RNA extraction kit (Takara, China) and stored at −80°C for use.

### Soluble expression and purification of Cas12a

The Cas12a was expressed and purified using the protocol with minor modifications (Mohanraju et al., 2018). The BL21(DE3) competent cells, transformed with expression plasmid 6 × His-MBP-TEV-huLbCpf1 (Addgene, 90,096), were inoculated with 200 mL LB medium until OD_600_ reached approximately 0.4 to 0.6 and then induced with 0.5 mM isopropyl-β-d-thiogalactopyranoside (IPTG; final concentration) for 8 h at 37°C. Cell pellets were harvested and resuspended in lysis buffer (150 mM NaCl, 50 mM Tris, 1 mM DTT, 1 mM EDTA, 1% Triton-X 100, 1× Protease Inhibitor Cocktail, 5 mM CHAPS, pH 7.4, Solarbio, Beijing, China). After sonication and centrifuge, the supernatant was collected and filtered through a 0.22 μm filter and further purified by nickel ion affinity chromatography using ÄKTA pure™ 150 L (GE Healthcare Life Sciences). Finally, Cas12a protein was eluted using elution buffers (1 M imidazole, 20 mM Tris–HCl, pH 8.0). Ultimately, the purified Cas12a protein was determined by SDS-PAGE and Western blot, then quantified using a BCA protein assay kit (Thermo Fisher Scientific, United States), and stored at −80°C for further study.

### Standard plasmid preparation

The fragment was generated from the FPV QD22-5 strain by PCR using primer pair FPV VP2-F/VP2-R (Sun et al., 2018). It was crucially cloned into the pMD™18-T vector (TaKaRa, Dalian, China) and sequenced to generate the recombinant plasmid pMD18-T-VP2. The recombinant plasmid was used as a standard in the RAA and PCR. The plasmids were quantified as described previously (Sun et al., 2018).

### Design and synthesis of primers, reporters, and crRNA

**Figure 2:**
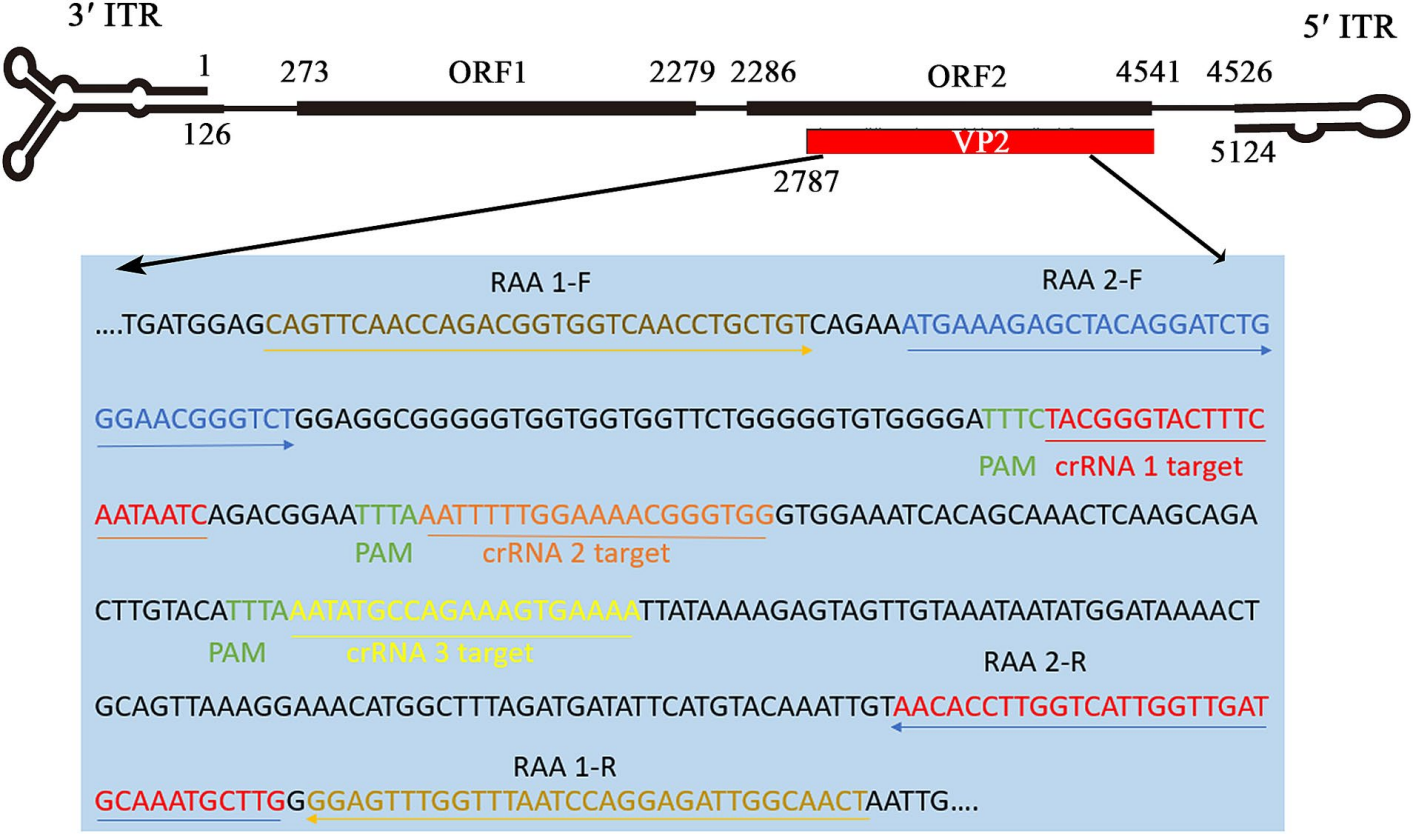
Binding sites of RAA primers and crRNA oligonucleotides targeting VP2 gene in FPV genome. According to the conserved sequence of the FPV strain (GenBank: M38246), two pairs of RAA primers and three crRNAs (crRNA1, crRNA2, crRNA3) were designed on the positive and negative chains of the FPV VP2 gene (2,787–4,541 nt), respectively.

The highly conserved region of the VP2 gene (nucleotides 6–388), detailed in the sequence alignment of the VP2 gene in the Supplementary Data Sheet 1, was chosen to design crRNA and RAA primer sequences. According to the principle of Cas12a, which explicitly recognizes PAM sequences (TTTN), we designed and synthesized three target sequences (crRNA 1, 2, and 3) for the target DNA. Two pairs of RAA primers were designed based on the above three crRNAs using Primer Premier 5.0 software (Figure 2). Two reporters, the fluorescent ssDNA reporter (reporter 1) and lateral flow strip test reporter (reporter 2) (Wei et al., 2022), primers, and crRNAs, were ordered from Comate Bioscience Co., Ltd. (Changchun, China). The oligonucleotide sequences of RAA primers, crRNAs, and the probes used in this study are shown in Table 1.

**Table 1:** Sequences of oligonucleotides used in this study.

Assay	Primers	Sequences (5′–3′)	Amplicon size
PCR	FPV 488-F	CAAATAGAGCATTAGGCTTACC	488 bp
	FPV 488-R	GTTAATTCCTGTTTTACCTCCA	
Plasmid	FPV VP2-F	ATGAGTGATGGAGCAGTTCA	1755 bp
	FPV VP2-R	TTAATATAATTTTCTAGGTGCTAG	
	RAA 1-F	CAGTTCAACCAGACGGTGGTCAACCTGCTGT	370 bp
RAA	RAA 1-R	AGTTGCCAATCTCCTGGATTAAACCAAACTCC	
	RAA 2-F	ATGAAAGAGCTACAGGATCTGGGAACGGGTCT	301 bp
	RAA 2-R	CAAGCATTTGCATCAACCAATGACCAAGGTGTT	
CRISPR/Cas12a	crRNA 1	*UAAUUUCUACUAAGUGUAGAU* UACGGGUACUUUCAAUAAUC	
	crRNA 2	*UAAUUUCUACUAAGUGUAGAU* AAUUUUUGGAAAACGGGUGG	
	crRNA 3	*UAAUUUCUACUAAGUGUAGAU* AAUAUGCCAGAAAGUGAAAA	
	ssDNA reporter 1	FAM-TTTATTT-BHQ1	
	ssDNA reporter 2	FAM-TTTTTTTATTTTTTT-Biotin	

### RAA reactions and PCR

DNA extraction, as described in our previous studies (Sun et al., 2018; Sun et al., 2019), was the first step in a thorough research process. RAA reactions were then performed using a kit (Jiangsu Qitian Gene Biotechnology Co., Ltd., Wuxi, China) according to the manufacturer’s protocol. Briefly, the reactions were performed in a total volume of 50 μL comprising 25 μL 2× buffer V, 2 μL of each primer (RAA-F/R, 10 μmol/L), 14 μL ddH_2_O, 2 μL of DNA input, and 5 μL magnesium acetate I. The subsequent CRISPR/Cas12a cleavage reaction was incubated at 37°C in a dry bath for 30 min. We also established a conventional PCR for FPV detection, a process that was equally thorough. The PCR was carried out in a 15 μL reaction volume containing 7.5 μL of 2× Es Taq Master mix, 1 μL each of the forward and reverse primers (FPV 488-F/R, 10 μmol/L), 1 μL of extracted DNA or standard plasmid, and 4.5 μL of ddH_2_O. The reaction conditions were as follows: 5 min at 98°C, followed by 35 cycles at 98°C for 40 s, 55.5°C for 30 s and 72°C for 45 s, and a final elongation at 72°C for 10 min.

### CRISPR/Cas12a/FLUOR assay

The CRISPR/Cas12a fluorescent based detection (CRISPR/Cas12a/FLUOR) assay was performed as described previously with minor modifications (Jirawannaporn et al., 2022). The RAA product (2 μL) was added to 20 μL of the CRISPR-Cas12a reaction mixture containing 2 μL NEBuffer 2.1, 0.2 μL RNase inhibitor, 3 μL crRNA (75 nmol/L), 0.8 μL Cas12a (40 nmol/L), 0.6 μL ssDNA reporter 1 (300 nmol/L), and 11.4 μL of RNase-free water. Then, the reactions were incubated in a Real-Time PCR Detection System (Roche, Mannheim, Germany) for up to 60 min at 37°C with fluorescent signals collected every 5 min (ssDNA FQ substrates = λex: 485 nm; λem: 520 nm). With respect to visual detection, the reactions were incubated in a dry bath at 37°C for 30 min.

### CRISPR/Cas12a/LFS assay

The total reaction system of CRISPR/Cas12a lateral flow strip-based detection (CRISPR/Cas12a/LFS) assay was made up to 20 μL. It consisted of 2 μL NEBuffer 2.1, 0.2 μL RNase inhibitor (TaKaRa, Dalian, China), 3 μL crRNA (75 nmol/L), 0.8 μL Cas12a, 0.2 μL ssDNA reporter 2 (100 nmol/L), 2 μL RAA amplification product and 11.8 μL ddH_2_O. The reaction system is added to the centrifuge tube, mixed, shaken, and centrifuged. The reaction mixture was incubated in a dry bath at 37°C for 30 min. After the reaction, 5 μL product was sucked out and mixed with 45 μL ddH_2_O in a new tube with spinning. The lateral flow dipstick (Tiosbio, Beijing, China) was inserted into the new tube with the pad end, and the liquid level should not exceed the top end of the pad. The test result can be read directly according to the color condition of the strip (Figure 1).

### Sensitivity and specificity of the assay

The RAA-CRISPR/Cas12a/LFS (FLUOR) assay was rigorously evaluated for its sensitivity and specificity. The standard plasmid pMD18-T-VP2 was diluted from 10^7^ to 10^0^ copies/μL, and the tenfold serial dilution of plasmid was meticulously used to test the limits of the assay. The specificity of the assay was assessed by testing FPV and other cat viruses, including FCoV (DF2 strain), FHV (BJ22-5 strain), and FCV (BJ22-11 strain). Viral RNA or DNA samples were extracted from infected cell cultures using the Takara MiniBEST Viral RNA/DNA Extraction Kit, as described previously (Sun et al., 2018). As described previously, RNA extraction of FCoV and FCV was transcripted into cDNA (Sun et al., 2018). The cDNA or DNA was assayed using the RAA-CRISPR/Cas12a/LFS (FLUOR) assay.

### Evaluation of the RAA-CRISPR/Cas12a/LFS assay using clinical samples

The performance of the RAA-CRISPR/Cas12a/LFS system was evaluated using 43 feline clinical samples. Briefly, 2 μL of feline clinical samples DNA were used for RAA amplification, and the procedure was completed in 30 min in a dry bath; 2 μL of RAA amplification product was added to the CRISPR/Cas12a detection system, which was completed in 30 min, and the result was indicated by lateral flow dipstick (RAA-CRISPR/Cas12a/LFS assay). Conventional PCR was employed to detect FPV DNA and to sequence some PCR products to confirm the results using different methods. Compared with conventional PCR, the percentage of agreement, relative sensitivity, and relative specificity of RAA-CRISPR/Cas12a/LFS assay were calculated according to the methods in a previous study (Wang et al., 2015).

## Results

### Expression and purification of Cas12a

**Figure 3:**
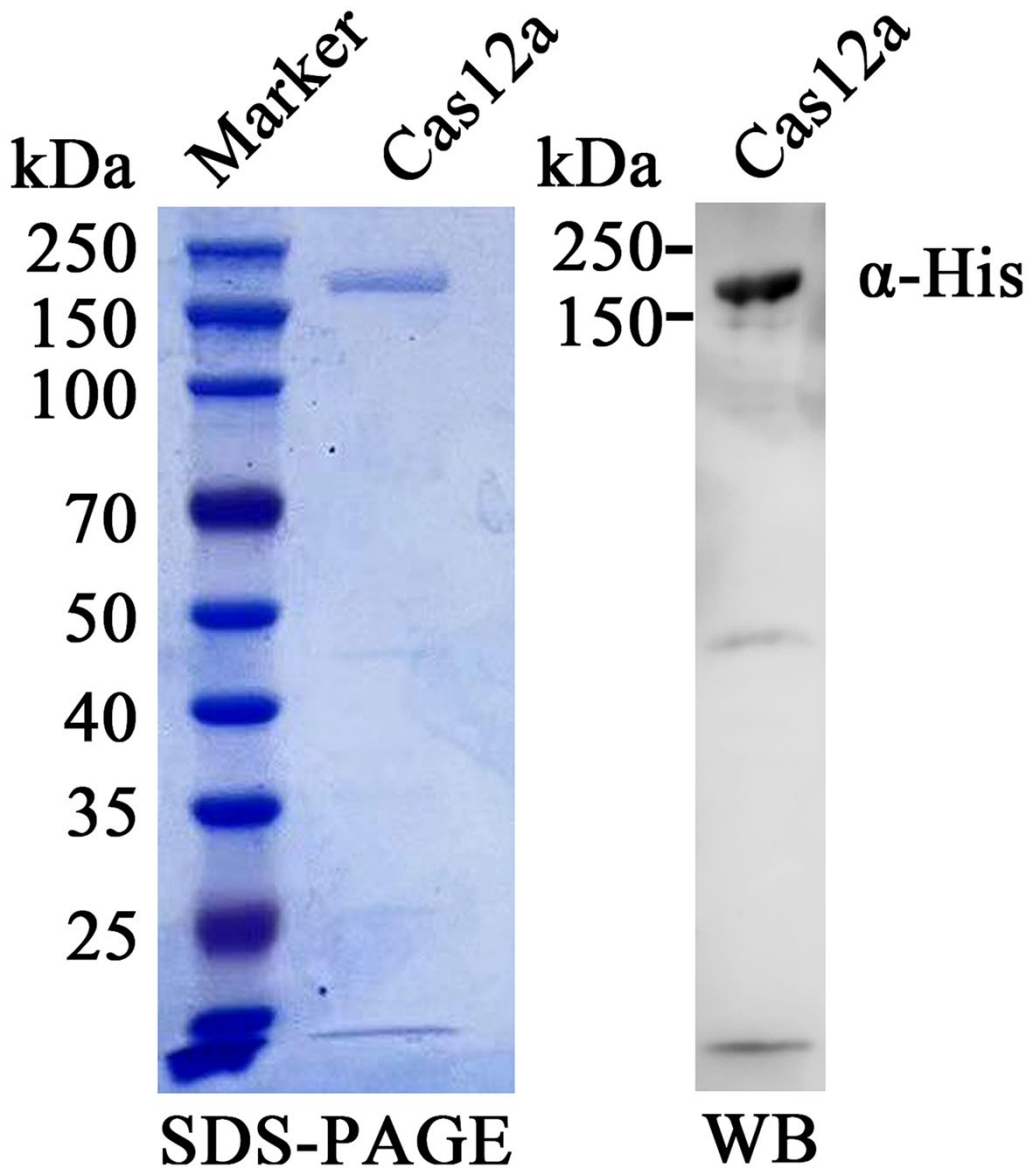
Purification and identification of expressed Cas12a protein. Recombinant Cas12a-MBP-His protein was purified, separated on an SDS-PAGE gel, and stained with Coomassie brilliant blue dye (left panel). The protein was probed using anti-His antibody and HRP-labeled goat anti-mouse IgG (right panel). The band of purified Cas12a protein was evident both in SDS-PAGE and Western blot (WB).

The results of Sodium Dodecyl Sulphate-Polyacrylamide Gel Electrophoresis (SDS-PAGE) showed that Cas12a protein was a mainly soluble expression, not in inclusion bodies. A single band with a molecular weight of about 192 kDa was obtained by SDS-PAGE and Western blot after nickel ion affinity chromatography (Ni-NTA Agarose) (Figure 3). A total of 5 mg purified Cas12a was obtained from 200 mL bacterial LB cultures.

### Design and screening of the RAA primers and crRNA

**Figure 4:**
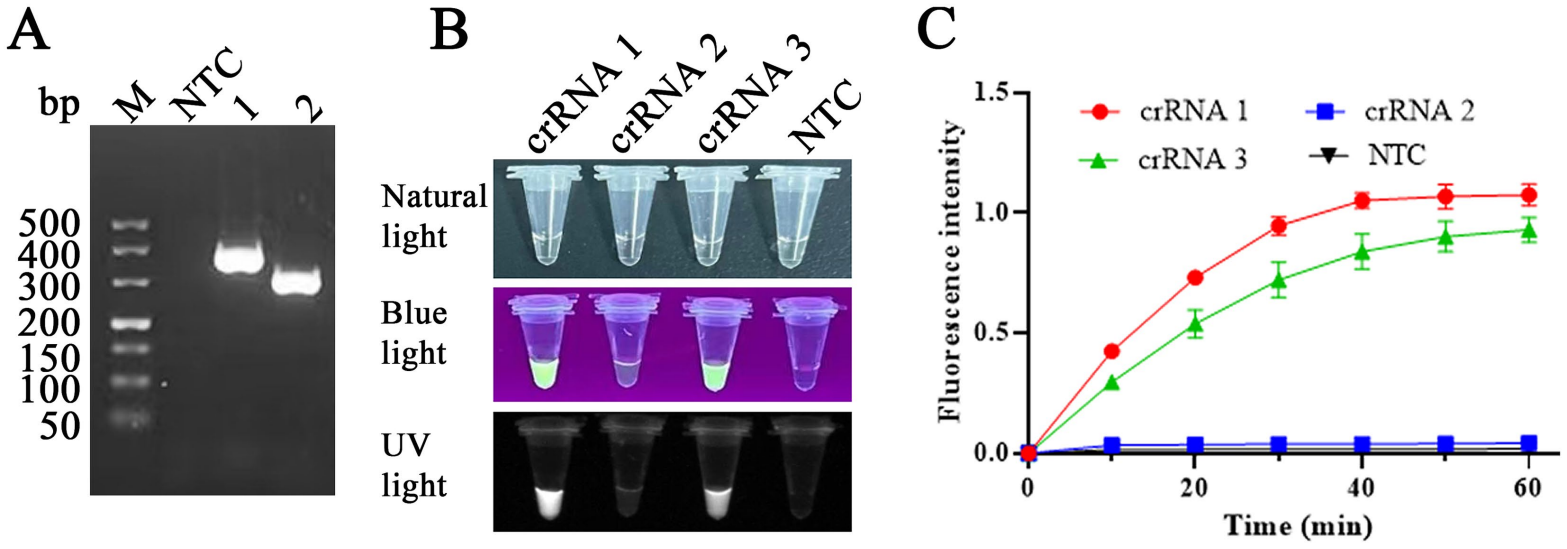
RAA primers and crRNA screening. **(A)** Primers RAA-1F/R (lane 1) and RAA-2F/R (lane 2) were used to amplify the VP2 gene, with fragments of 370 and 301 bp, respectively, in the RAA assay. NTC: non-target control. **(B,C)** Among three candidate crRNAs for FPV, crRNA 1 produced the highest fluorescent signals, exhibiting the ability to recognize FPV VP2 DNA. Primer RAA-2F/R and crRNA1 were selected for RAA assay in this study.

The RAA reaction was performed at 37°C for 30 min to screen the optimal RAA primers, and then the products were evaluated on the electrophoretic gel. Two RAA primer pairs (Table 1) with specific fragment lengths of 370 and 301 bp (Figure 4A), respectively, were compared, and based on gel quantification analysis by ImageJ 1.46r software and the sizes of product, primers RAA 2-F/R were selected for use in RAA assay. Three crRNAs (Table 1) were designed and used to perform CRISPR/Cas12a visual test. We use an LC96 fluorescence quantitative PCR instrument and the naked eye under blue light to evaluate the crRNA-guided Cas12a cleavage activity. Among three candidate crRNAs for FPV, crRNA 1 produced the highest fluorescent signals, exhibiting the ability to recognize the FPV VP2 gene and activate Cas12a efficiently (Figures 4B,C). crRNA 1 was considered the best and selected for use in the CRISPR/Cas12a detection assay.

### RAA and PCR for FPV detection

**Figure 5:**
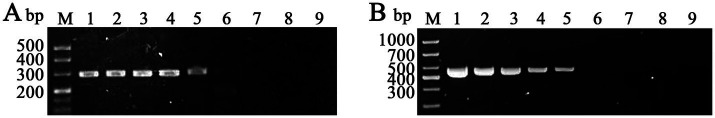
Evaluation of the sensitivities of RAA and conventional PCR for the detection of PFV VP2 plasmid DNA. M: DNA marker; lanes 1–8: different FPV VP2 plasmid DNA copies subjected to RAA **(A)** and conventional PCR **(B)** (2.1 × 10^7^, 2.1 × 10^6^, 2.1 × 10^5^, 2.1 × 10^4^, 2.1 × 10^3^, 2.1 × 10^2^, 2.1 × 10^1^, and 2.1 × 10^0^ copies/μL, respectively); lane 9: blank.

Using the primers RAA 2-F/R selected above and FPV 488-F/R for RAA and PCR, respectively, we characterized the sensitivity of both assays with serial tenfold dilutions of the recombinant plasmid pMD18-T-VP2 (ranging from 2.1× 10^7^ to 2.1 × 10^0^ DNA copies/μL). The detection limits for the RAA and conventional PCR are 2.1 × 10^3^ DNA copies per reaction for both assays (Figure 5).

### Optimization of CRISPR/Cas12a

**Figure 6:**
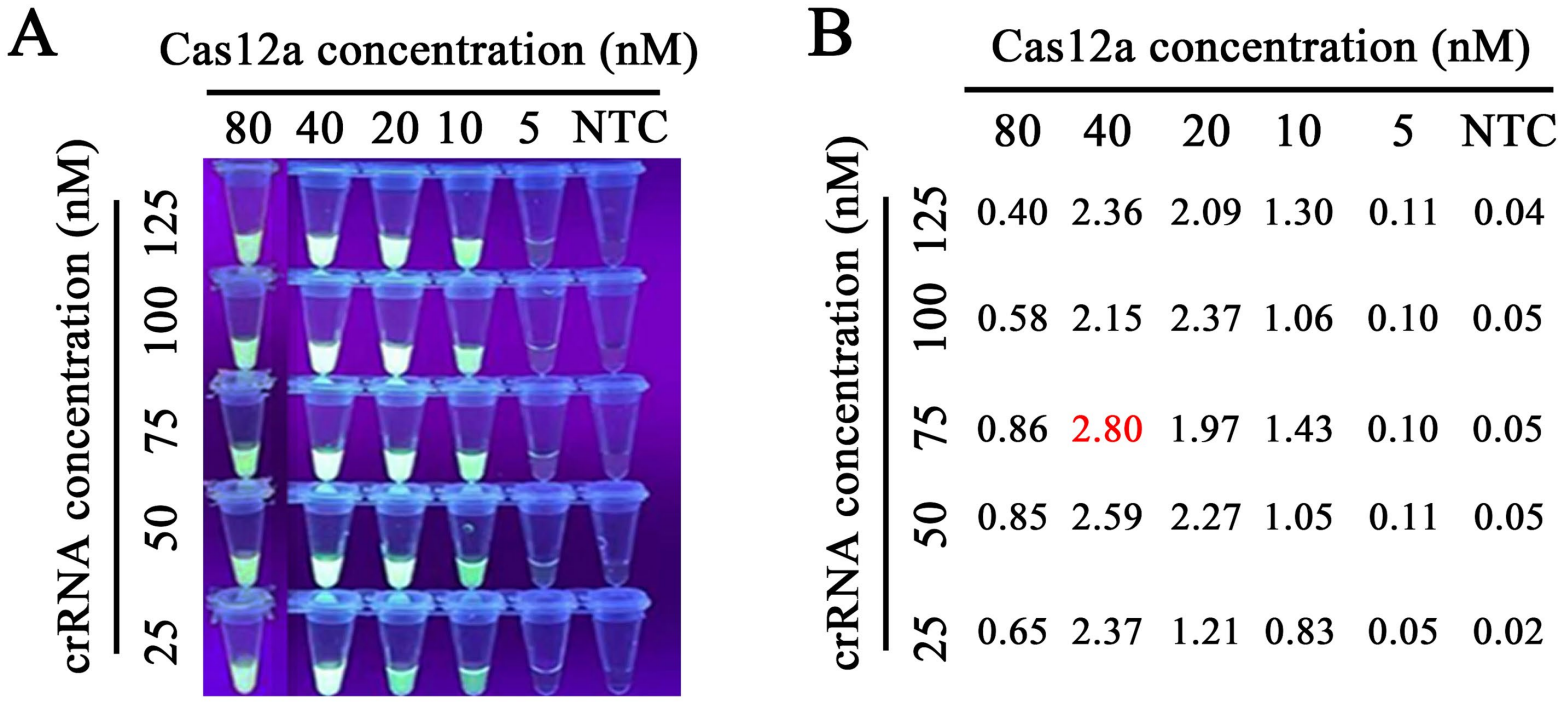
Optimization of Cas12a protein and crRNA concentrations. Our research involved the use of gradient concentrations of Cas12a protein (5, 10, 20, 40, and 80 nM) and crRNA (25, 50, 75, 100, and 125 nM) for a titration test. This meticulous process led us to the precise determination of the optimal concentrations in the RAA-CRISPR/Cas12a reaction system. We found that 40 nM of Cas12a protein and 75 nM crRNA1 were the optimal concentrations for RAA detection, as indicated by the highly accurate fluorescence brightness **(A)** and the fluorescence intensity **(B)** under blue light and Real-time PCR instrument, respectively.

After purifying the Cas12a protein and selecting crRNA above, we optimized the concentrations of Cas12a protein and crRNA in the Cas12a-mediated fluorescence detection assay, as the concentrations of Cas12a and crRNA play a crucial role in the detection of fluorescence. The checkerboard method determined five different concentration gradient values of Cas12a protein (5, 10, 20, 40, and 80 nM) and crRNA (25, 50, 75, 100, and 125 nM). As shown in Figure 6, the results showed that all concentration values had fluorescence signal and, relatively, the concentration of Cas12a protein at 40 nM and that of crRNA at 75 nM had higher fluorescence intensity than that of other concentration values at the emission wavelength of 510 nm determined by an LC96 fluorescence quantitative PCR instrument. Thus, 40 nM of Cas12a protein and 75 nM of crRNA were optimal. In the CRISPR/Cas12a/FLUOR assay, the ssDNA reporter 1 (FAM-TTTATTT-BHQ1) was trans-cleaved by Cas12a protein, and then the fluorescence of FAM was measured by a Real-Time PCR Detection System or can be visible by the naked eye under the blue light. In the CRISPR/Cas12a/LFS assay, the ssDNA reporter 2 (FAM-TTTTTTTATTTTTTT-Biotin) was trans-cleaved by Cas12a protein and then detected by commercially available dedicated nucleic acid test strips, with reading the results result directly according to the color development of the test strip. The positive of FPV will read the T test line red, or both the T and C test lines red, while the negative of FPV will read the C test line red only (Figure 1).

### Sensitivity of RAA-CRISPR/Cas12a

**Figure 7:**
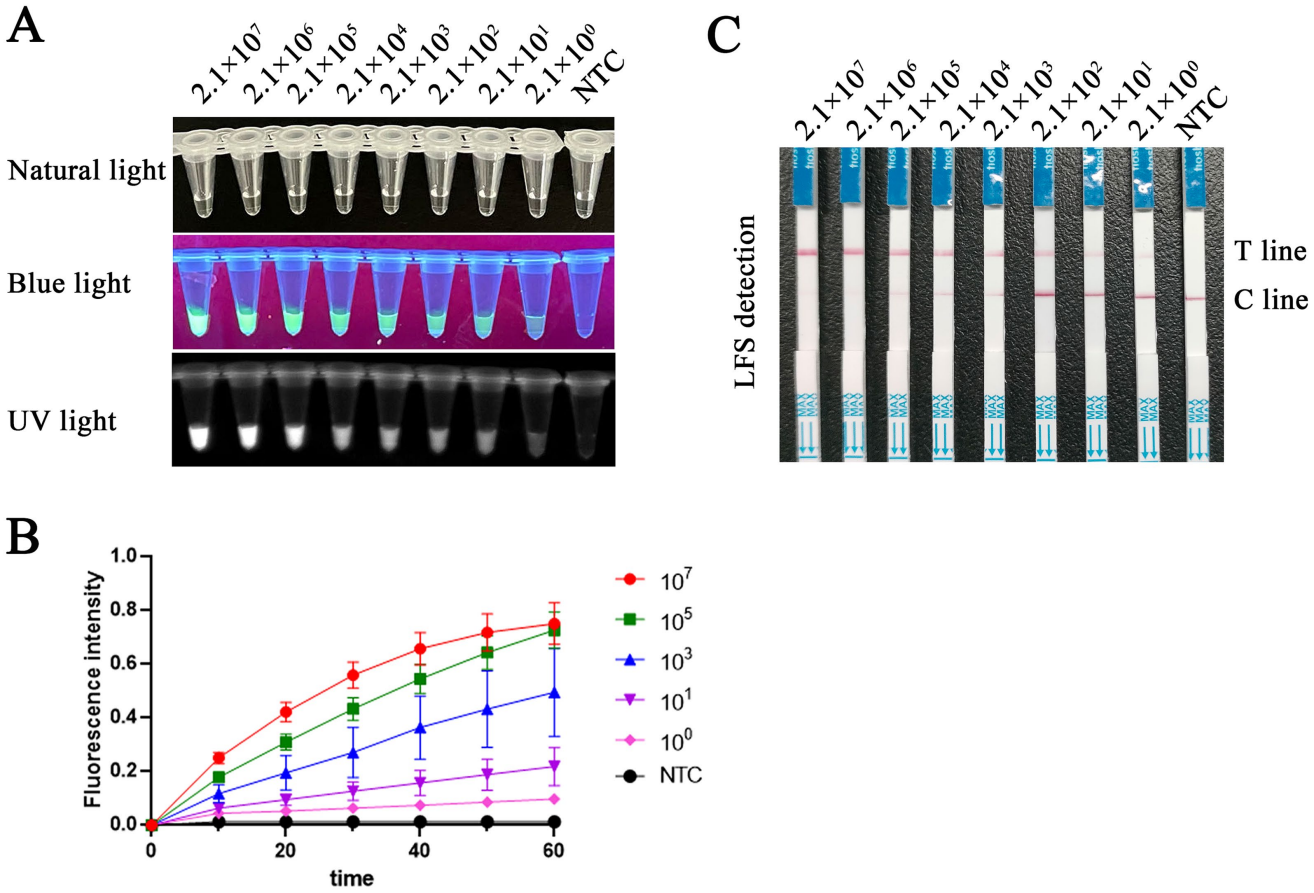
Sensitivity comparison between RAA-CRISPR/Cas12a/FLUOR and RAA-CRISPR/Cas12a/LFS. Tenfold gradient dilutions of FPV VP2 plasmid DNA copies (2.1 × 10^7^ to 2.1 × 10^0^ copies/μL) subjected to RAA-CRISPR Cas12a/FLUOR **(A,B)** and RAA-CRISPR/Cas12a/LFS **(C)** sensitivity validation. ddH_2_O was used as a control in the NTC group. The CRISPR Cas12a/FLUOR and CRISPR/Cas12a/LFS detection limits are 2.1 copies per reaction.

To evaluate the detection sensitivity of RAA-CRISPR/Cas12a/LFS (FLUOR), the plasmid pMD18-T-VP2 was diluted from 2.1 × 10^7^ to 2.1 × 10^0^ copies/μL and was used as a template. The detection sensitivity of the RAA-CRISPR/Cas12a/LFS assay was determined. We found that the detection limit of PCR for the plasmid pMD18-T-VP2 was only 2.1 × 10^3^ copies per reaction as shown above. As shown in Figure 7, the detection sensitivity of RAA-CRISPR/Cas12a/LFS (FLUOR) for the plasmid pMD18-T-VP2 was 2.1 × 10^0^ copies per reaction. The results showed that the RAA-CRISPR/Cas12a/LFS (FLUOR) method was more sensitive to detecting FPV than the conventional PCR method.

### Specificity of RAA-CRISPR/Cas12a

**Figure 8:**
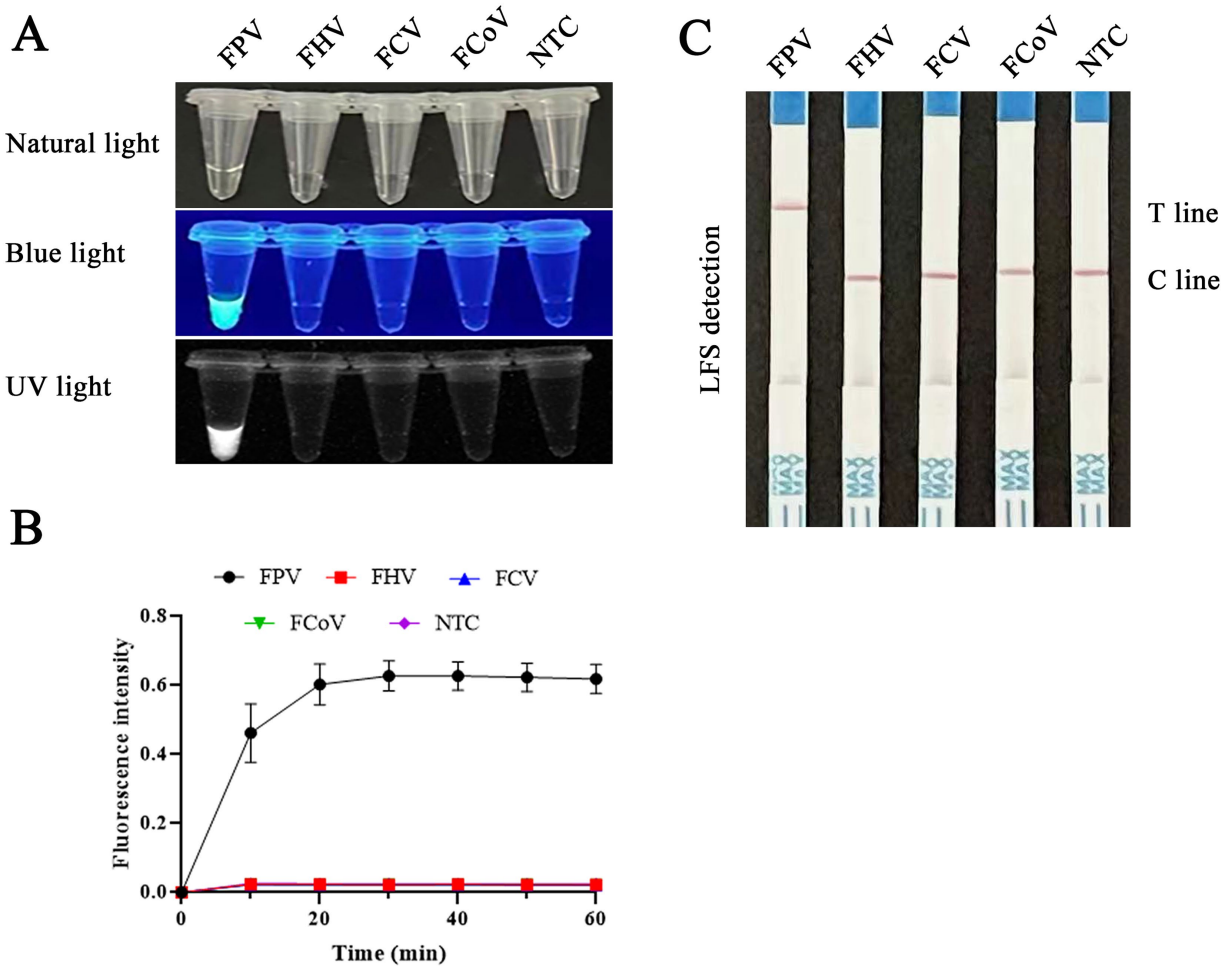
Evaluation of the specificity of RAA-CRISPR/Cas12a/FLUOR and RAA-CRISPR/Cas12a/LFS. Product detection using CRISPR/Cas12a/FLUOR and CRISPR/Cas12a/LFS. RAA was used to detect DNA or cDNA of FPV, FHV, FCV, and FCoV. Then the products were detected by CRISPR/Cas12a/FLUOR **(A,B)** and CRISPR/Cas12a/LFS **(C)**.

Our assessment of the detection specificity of the RAA-CRISPR/Cas12a/LFS (FLUOR) assay, using viral DNA or cDNA from FPV, FCoV, FHV, and FCV, yielded promising results. As shown in Figure 8, only the FPV samples produced positive bands on the test line. In contrast, the other virus samples and negative controls consistently showed negative results, with only the quality control line displaying bands. The RAA-CRISPR/Cas12a/LFS assay detected only the FPV samples with a fluorescence signal and no signals for other viruses and negative samples. Importantly, there was no cross-reaction with other feline pathogens, confirming the high specificity of the RAA-CRISPR/Cas12a/LFS (FLUOR) assay.

### Comparison of RAA-CRISPR/Cas12a/LFS and PCR

**Figure 9:**
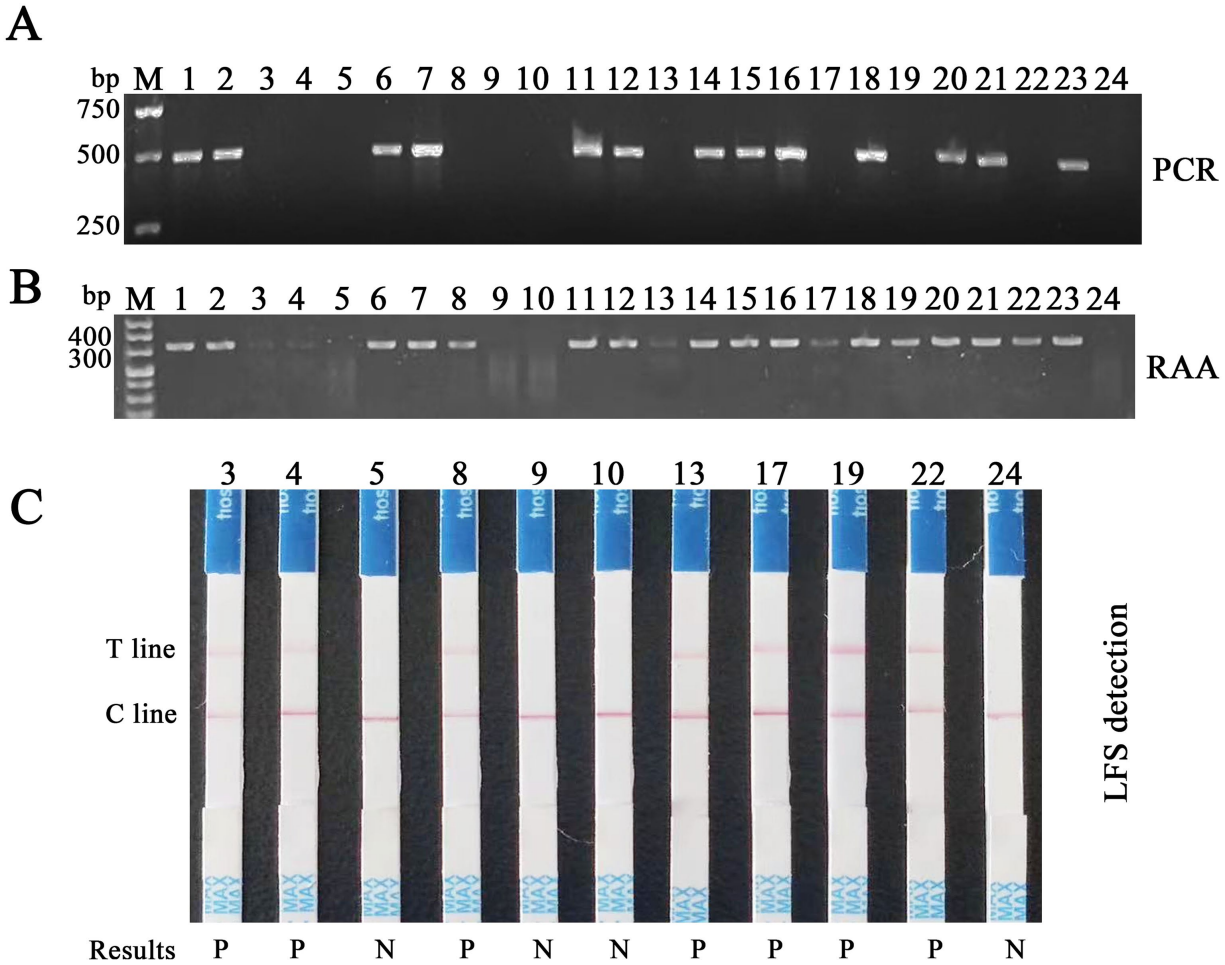
Detection of feline clinical samples by RAA-CRISPR/Cas12a/LFS and PCR. **(A)** Results of PCR detection of the first batch of 24 feline clinical samples, with 13 positives (Nos. 1, 2, 6, 7, 11, 12, 14–16, 18, 20, 21, and 23) and 11 negatives (Nos. 3–5, 8–10, 13, 17, 19, 22, and 24). **(B)** Results of RAA detection of the same samples, with 20 positives (Nos. 1–4, 6–8, and 11–23) and four negatives (Nos. 5, 9, 10, and 24). **(C)** Results of RAA-CRISPR/Cas12a/LFS detection of 11 clinical samples, which are negative tested by PCR, with seven positives (Nos. 3, 4, 8, 13, 17, 19, and 22) and four negatives (Nos. 5, 9, 10, and 24), highlighting the high accuracy and reliability of this method. Results show that P means positive and N means negative.

RAA-CRISPR/Cas12a/LFS and PCR were utilized to identify FPV in clinical samples, with a comprehensive evaluation of the clinical effectiveness of RAA-CRISPR/Cas12a/LFS in detecting FPV (Figure 9). The RAA-CRISPR/Cas12a/LFS detection was conducted on 43 feline clinical samples, as meticulously detailed in Table 2. The detection rate of the RAA-CRISPR/Cas12a/LFS assay was 46.5% (20/43), which was significantly higher than that of the PCR assay (13/43, 30.2%). The percentage of agreement, relative sensitivity, and relative specificity of the RAA-CRISPR/Cas12a/LFS assay were 83.7% (36/43), 100% (13/13), and 76.7% (23/40), respectively (Table 2). From the clinical samples that tested positive for FPV by RAA-CRISPR/Cas12a/LFS and PCR, five FPV isolates were sequenced and deposited in GenBank with Nos. OR727315, OR727316, OR727317, OR727318, and OR727319. This thorough research process instills confidence in the results and their implications.

**Table 2:** Comparison of CRISPR/Cas12a/LFS and PCR results for clinical samples.

CRISPR/Cas12a/LFS	Conventional PCR		
	Positive	Negative	Total
Positive	13	7	20
Negative	0	23	23
Total	13	30	43

Percentage of agreement: (13 + 23)/43 = 83.7%; relative sensitivity: 13/(13 + 0)/ = 100%; relative specificity: 23/(23 + 7)/ = 76.7%.

## Discussion

Feline panleukopenia, first described in France in 1928, is a disease that affects domestic and wild cats and other animals globally. FPV is not known to infect humans but was isolated from monkeys in a Chinese outbreak, which may threaten humans in the future (Yang et al., 2010; Sykes and Parrish, 2021). This highlights the global impact of these diseases and the need for more sensitive and specific methods for cat parvovirus detection. Canine parvovirus (CPV), derived from a virus related to FPV, caused a worldwide pandemic of illness in dogs in 1978. The original type, CPV-2, did not infect cats but was subsequently replaced by three antigenic variants, CPV-2a, CPV-2b, and CPV-2c, which can infect cats and cause feline parvoviral enteritis (Wang et al., 2016; Sun et al., 2019). Devastating outbreaks of feline parvoviral enteritis usually lead to high mortalities of large cats, especially in some animal shelters. This underscores the urgent need for more sensitive and specific methods for cat parvovirus detection.

Serval microbiological methods were used to detect FPV. ELISA was used to detect FPV antigens from feces or rectal swabs, whose sensitivity and specificity vary from one to another. False-negative results are commonly found in ELISAs (Neuerer et al., 2008; Abd-Eldaim et al., 2009). Virus isolation was also used to identify FPV from feline clinical samples. However, this assay was a complicated procedure and was widely used in scientific research labs, but not widely available in clinics (Wang et al., 2016; Zhao et al., 2022). Electron microscopy is another specialized procedure used in many laboratories to identify FPV, However, its sensitivity is poor, so this test is not typically used for routine diagnosis (Wang et al., 2012). The limitations of each method suggest a need for the development of more reliable, sensitive, and practical diagnostic techniques for FPV in veterinary medicine. This would aid in early and accurate diagnosis, which is crucial for effective treatment and control of FPV-related diseases in cats.

Nucleic acid-based molecular diagnostic tests, including FPV, were widely used for parvovirus detection. We have developed a nanoparticle-assisted PCR (nanoPCR) assay for detecting carnivore parvoviruses, specifically FPV, CPV, and mink enteritis virus (MEV) (Wang et al., 2015). Based on the SNPs in the VP2 gene between the original type CPV-2 and the variants (CPV-2a, 2b, and 2c), Sun et al. designed two pairs of primers and five probes and established a multiplex TaqMan real-time PCR assay that detected effectively and differentiated CPV-2, 2a, 2b and 2c by two separate real-time PCRs (Sun et al., 2018). Combining the conserved segment and one SNP detection between CPV and FPV, Sun et al. developed a high-resolution melting analysis assay that simultaneously distinguishes CPV from FPV in clinical samples (Sun et al., 2019). Loop-mediated isothermal amplification (LAMP) is another molecular diagnosis using nucleic acid-based testing, which was wildly used to detect viruses, bacteria, and other pathogens (Lai et al., 2024; Pang et al., 2024; Peng et al., 2024; Wang H. et al., 2024). We applied LAMP for the detection and differentiation of wild-type and vaccine strains of MEV in our lab, and these assays are sensitive, easy, and less time-consuming methods (Wang et al., 2013; Lin et al., 2018).

Recombinase-aided amplification and recombinase polymerase amplification (RPA) are isothermal nucleic acid amplification technologies that can adapt to different contexts. They use a cocktail of recombinase enzymes, single-stranded binding (SSD) proteins, and DNA polymerases. These technologies have been successfully integrated with a variety of detection strategies, from real-time fluorescent detection to end-point lateral flow strips (Lobato and O'Sullivan, 2018). The amplification products of RAA or RPA can be directly visualized by agarose gel electrophoresis or combined with a fluorescence probe and lateral flow strip, detected by real-time thermocycler and naked eye without any devices (Zeng et al., 2022; Yang et al., 2023; Zhu et al., 2023). The CRISPR/Cas system, a novel nucleic acid detection technology, can be combined with isothermal nucleic acid amplification technologies, like LAMP, RAA, RPA, and so on, for widely used viruses detection (Xiong et al., 2020; Zhang et al., 2021; Wei et al., 2022; Zhu et al., 2022; Lei et al., 2023; Wang Y. et al., 2024). In a previous study, Wang T. et al. (2024) established an RPA-CRISPR/Cas12a-based real-time or end-point fluorescence detection method to identify feline parvovirus, in which they only combined with a fluorescence probe but not using a lateral flow strip. And in another paper, Wang et al. (2019) established a lateral flow dipstick RPA for the detection of FPV, in which they combined RPA and lateral flow dipstick without CRISPR/Cas system. Neither of the two papers claims that the methods can be used for CPV detection or not in their documents.

In this study, we developed an RAA-CRISPR/Cas12a platform to detect FPV. The results can be visualized by agarose gel electrophoresis after RAA application or combined with CRISPR/Cas12a to cut two types of reporters. This can be especially useful when utilizing a lateral flow strip, detected by a real-time thermocycler and naked eye without the need for any devices (Figure 1). This method can be applied for on-site detection. Additionally, we established a PCR method for feline parvovirus detection in this study. The RAA reaction only requires incubation at 37°C in a dry bath for 30 min. In contrast, the PCR reaction needs to be on a gene amplification machine for 82 min. Both the amplified fragments from RAA and PCR can be analyzed using agarose gel electrophoresis. Furthermore, aside from agarose gel electrophoresis, the RAA products can also be detected using CRISPR/Cas12a/FLUOR and CRISPR/Cas12a/LFS after the Cas12a reaction at 37°C for 30 min. Therefore, RAA proves to be a more efficient method compared to PCR in this study. Cas12a is an endonuclease that creates a double-strand break (DSB) at specifically targeted sites within the genome (Zetsche et al., 2015), after which it can trans-cleavage cut ssDNA reporters. In the protein expression experiment, we transformed the plasmid 6 × His-MBP-TEV-huLbCpf1 into *Escherichia coli* BL21(DE3) and purified the Cpf1 protein (now referred to as Cas12a), and then the protein was used for subsequent detection experiments. We compared the activity of Cas12a protein (Cat No.: M0653T) from NEB and that of purified Cas12a proteins in the trans-cleavage fluorescent ssDNA reporter (FAM-TTTATTT-BHQ1) and got equal fluorescent signals by a Real-Time PCR Detection System. Compared to wild Cas12a protein, the Cpf1 protein fused with maltose binding protein (MBP) tag was used in *in vitro* cleavage assay without cut MBP by TEV protease and found the MBP tag did not affect the endonuclease activity. The results are similar to previous research (Zhang et al., 2023). DNA extraction is the first and critical step for detection based on nucleic acid amplification technologies. Not only was the DNA extraction kit used to extract parvovirus DNA, but we also used water boiling to extract parvovirus. In the latter, the stool samples were mixed with PBS in a ratio of 1:1 (mass: volume) and heated at 95°C in a dry bath for 5 min, then centrifugated, and was used for RAA and PCR assays, which was time-saved than that with DNA extraction kit. We analyzed 817 available full-length CPV and FPV VP2 gene sequences from GenBank (Supplementary Data Sheet 1). We found that they are highly conserved in the region of crRNA recognized in this study, so the CRISPR/Cas12a-based RAA-lateral flow strip and fluorescence can detect FPV and CPV (Supplementary Figure S1). Because FPV and new variants of CPV could infect cats and induce animals to show the same clinical symptoms, the methods were more useful for feline parvovirus detection than the two previous methods in the cat clinic (Wang et al., 2019; Wang T. et al., 2024).

In conclusion, we have developed a novel method for detecting FPV using CRISPR/Cas12a. This method incorporates both lateral flow strips and fluorescence, making it suitable for use in both laboratory and clinical settings.

## Data Availability

The datasets presented in this study can be found in online repositories. The names of the repository/repositories and accession number(s) can be found in the article/Supplementary material.

## Supplementary material

SUPPLEMENTARY FIGURE S1 Shows that RAA-CRISPR/Cas12a/LFS could detect CPV in clinical samples.

SUPPLEMENTARY DATA SHEET 1 Aligned VP2 gene sequences of CPV and FPV from GenBank.
