# Supplementary figures and images for: Rapid detection of feline parvovirus using RAA-CRISPR/Cas12a-based lateral flow strip and fluorescence
Source: Front Microbiol. 2025 Mar 11;16:1501635. doi: 10.3389/fmicb.2025.1501635 (PMC11932995; doi:10.3389/fmicb.2025.1501635)

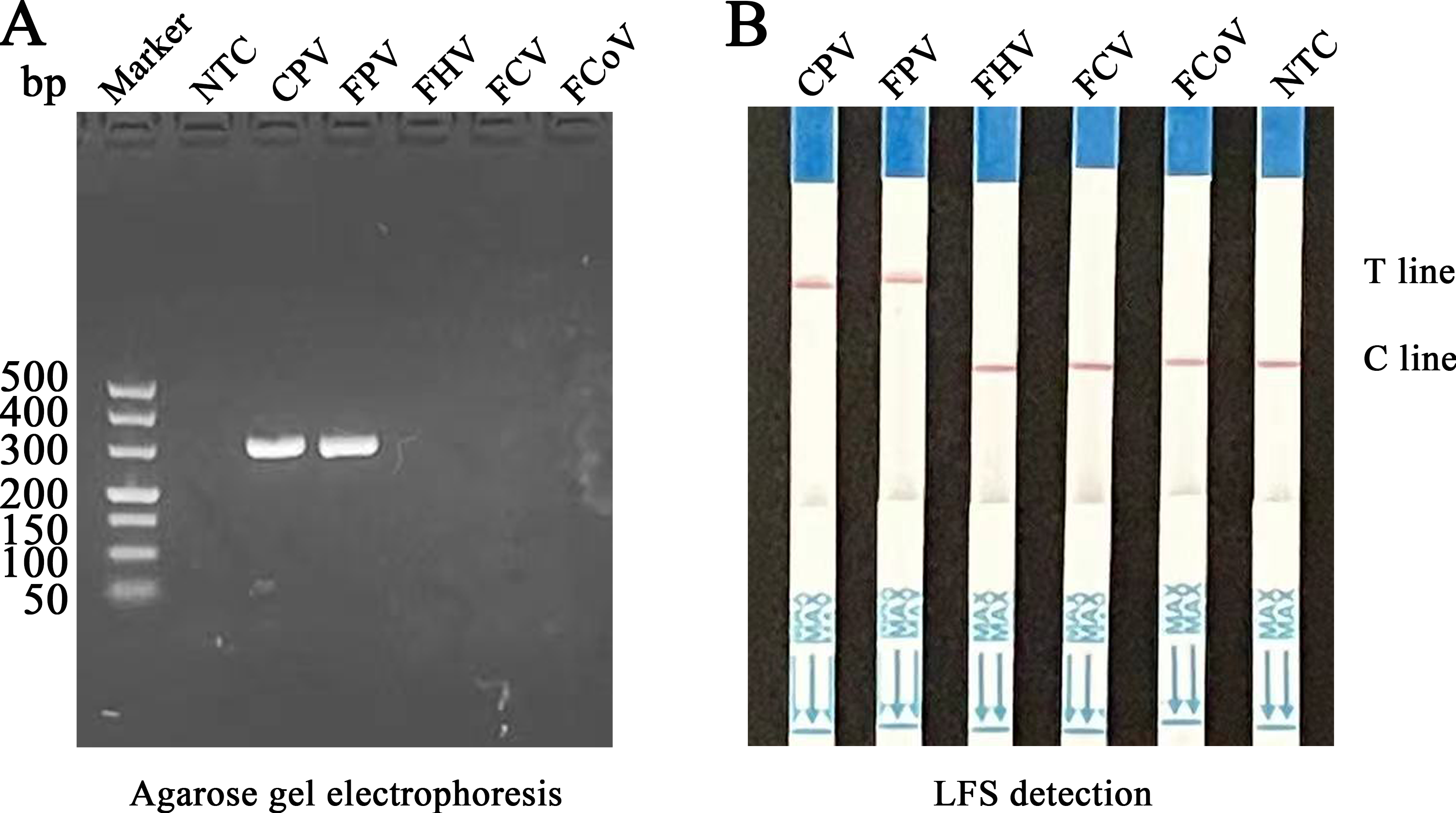

Supplement: SUPPLEMENTARY FIGURE S1 — Shows that RAA-CRISPR/Cas12a/LFS could detect CPV in clinical samples. [file Image_1.tif]
